# Endoscopic Excision of a C5 Osteoid Osteoma With Intraoperative Computed Tomography

**DOI:** 10.7759/cureus.117396

**Published:** 2026-10-03

**Authors:** Takahito Shimbo, Noriaki Yokogawa, Satoshi Kato, Masafumi Kawai, Satoru Demura

**Affiliations:** 1 Department of Orthopedic Surgery, Graduate School of Medical Sciences, Kanazawa University, Kanazawa, JPN

**Keywords:** cervical spine tumor, intraoperative ct imaging, microendoscopic excision, minimally invasive spine surgery, osteoid osteoma

## Abstract

Osteoid osteoma is a benign bone tumor characterized by nocturnal pain, with a predilection for teenage male patients. A 15-year-old boy presented with a two-month history of neck pain and was referred to our hospital, where imaging studies revealed an osteoid osteoma of the cervical vertebra. As he was preparing for a national basketball tournament and wished to return to sports as soon as possible, minimally invasive surgery was performed to excise the tumor using an endoscopic technique with intraoperative computed tomography, which confirmed that the extent of excision matched the preoperative surgical plan. His pain improved immediately postoperatively, and he was able to return to sports three months after surgery. At two years postoperatively, follow-up imaging showed no recurrence, progressive spinal malalignment, or spondylolisthesis.

## Introduction

Osteoid osteoma is a benign bone tumor that has a predilection for long bones, typically presenting with characteristic nocturnal pain relieved by nonsteroidal anti-inflammatory drugs (NSAIDs) in adolescent and young adult male patients. On computed tomography (CT), osteoid osteoma typically appears as a small radiolucent nidus with surrounding reactive sclerosis, whereas MRI may show prominent bone marrow and soft-tissue edema [1]. Osteoid osteoma is less common in the vertebrae (10%), and when it occurs in the spine, it is more frequently found in the lumbar and thoracic vertebrae than in the cervical spine [1]. Standard treatment options include NSAIDs as first-line therapy, with radiofrequency ablation (RFA) or surgical excision considered when conservative management fails [1,2]. The cervical spine presents unique anatomical challenges given its close proximity to the spinal cord and vertebral artery, and excessive excision of the posterior elements, particularly the facet joints, has been shown to increase segmental instability [3]. Endoscopic surgery has been reported to minimize tissue damage and reduce postoperative pain and deformity compared with open surgery [4], but its narrow field of view can result in incomplete excision of the tumor or excessive excision of the articular process [5,6]. In this report, we describe a case in which intraoperative CT, combined with endoscopic excision, enabled precise localization and confirmation of adequate tumor excision for an osteoid osteoma in the cervical vertebra.

## Case presentation

A 15-year-old boy presented to a referring physician with left posterior neck pain that worsened with movement. He was treated with loxoprofen 60 mg as needed at that physician's initial visit; the pain was relieved by NSAIDs but recurred after discontinuation, without particular nocturnal predominance. Because the pain did not improve after approximately two months of treatment, he was referred to our hospital for further evaluation. On physical examination, he had pain on motion of the left posterior neck without neurological deficit.

Plain radiographs, CT, MRI, and bone scintigraphy were obtained (Figure 1). Radiographs showed mild cervical kyphosis (C2-C7 Cobb angle, 12.5°) (Figures 1A, 1B). CT demonstrated a 12-mm lucent lesion with a central 2-mm nidus, surrounded by reactive sclerosis, confined to the inferior articular process of the left C5 lateral mass, located 3 mm from the spinal cord and medial to the transverse foramen containing the vertebral artery at its closest point (Figure 1C). The lesion did not extend into the adjacent neural foramen. MRI showed bone marrow edema and adjacent soft tissue signal change around the lesion (Figure 1D). Bone scintigraphy showed focal increased uptake at the corresponding site (Figure 1E). Based on these imaging findings, the differential diagnosis included osteoid osteoma, osteoblastoma, osteomyelitis, and aneurysmal bone cyst, and histological confirmation was considered important. Considering the physical examination findings and clinical course, osteoid osteoma was considered the most likely diagnosis.

**Figure 1:**
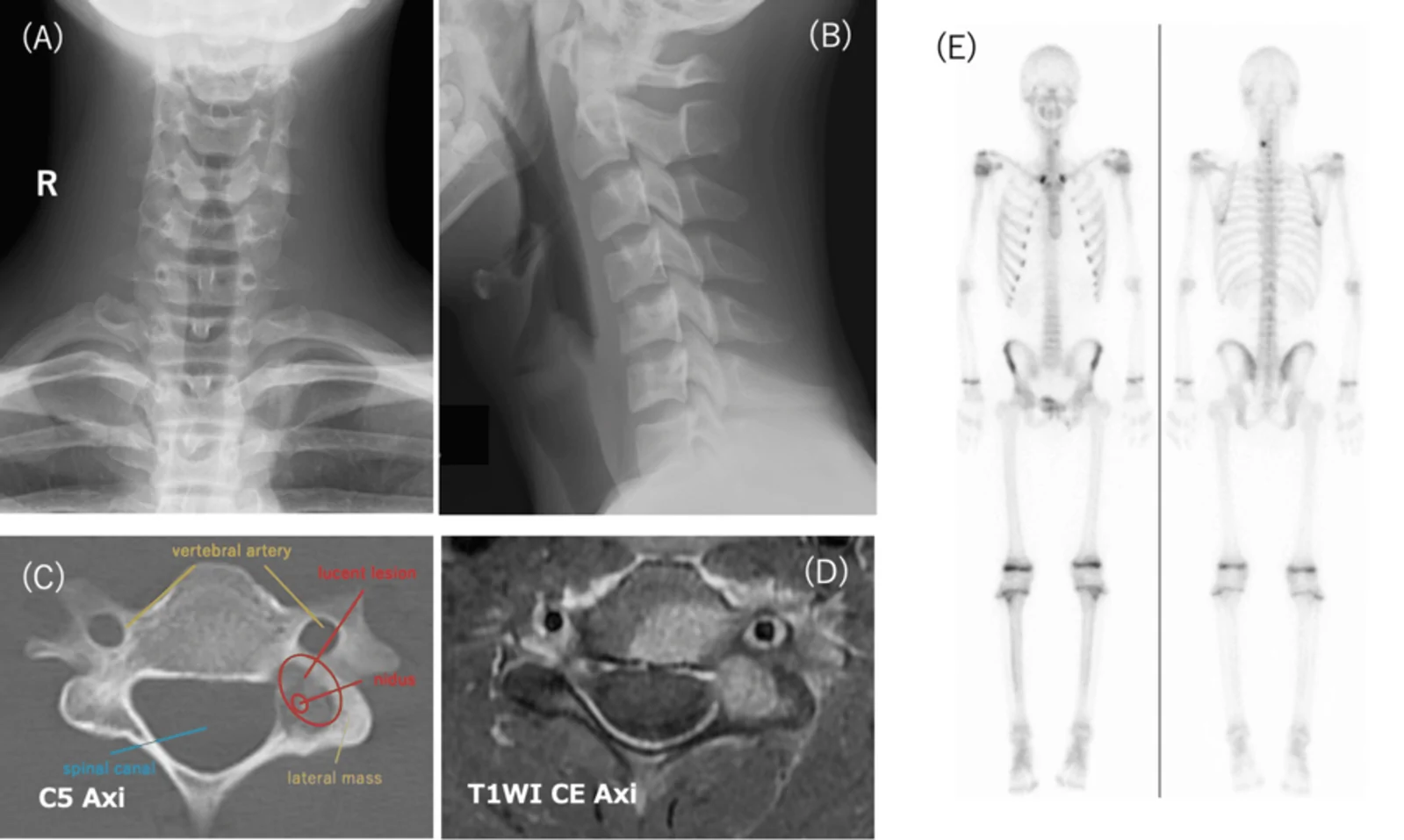
Preoperative imaging (A,B) Cervical radiographs showing mild cervical kyphosis (C2-C7 Cobb angle, 12.5°). (C) Axial CT showing a lucent lesion with a central nidus confined to the left C5 lateral mass; the spinal canal and vertebral artery are also labeled. (D) MRI showing bone marrow edema and soft tissue signal change in the same area. (E) Bone scintigraphy showing focal increased uptake in the same area CT: computed tomography

RFA was not considered because the lesion was located only 3 mm from the spinal cord. Percutaneous radiofrequency (RF) electrodes are generally recommended to remain at least 1 cm from major neural structures, and most spinal lesions closer than this margin have been considered unsuitable for this technique [7]. More recent reports have suggested that RFA may be performed safely closer to neural structures using intraoperative motor-threshold monitoring, but this technique was not used in our case [8]. Given the differential diagnosis above and the importance of histological confirmation, endoscopic excision (METRx System, Medtronic Sofamor Danek, Inc., Minneapolis, MN) was chosen, as it allows direct visualization of the lesion while obtaining a specimen for histological diagnosis. Because the patient was young and hoped to return to sports as soon as possible, this minimally invasive approach was also considered advantageous over open surgery.

Surgery was performed with the patient in the prone position. No dedicated navigation system was used; localization relied on preoperative CT for surgical planning and intraoperative CT. Approximately 10 mm of the left C5 lamina was excised under direct endoscopic visualization to access the lateral mass, and the tumor was approached from the left side of the C5 spinous process (Figure 2).

**Figure 2:**
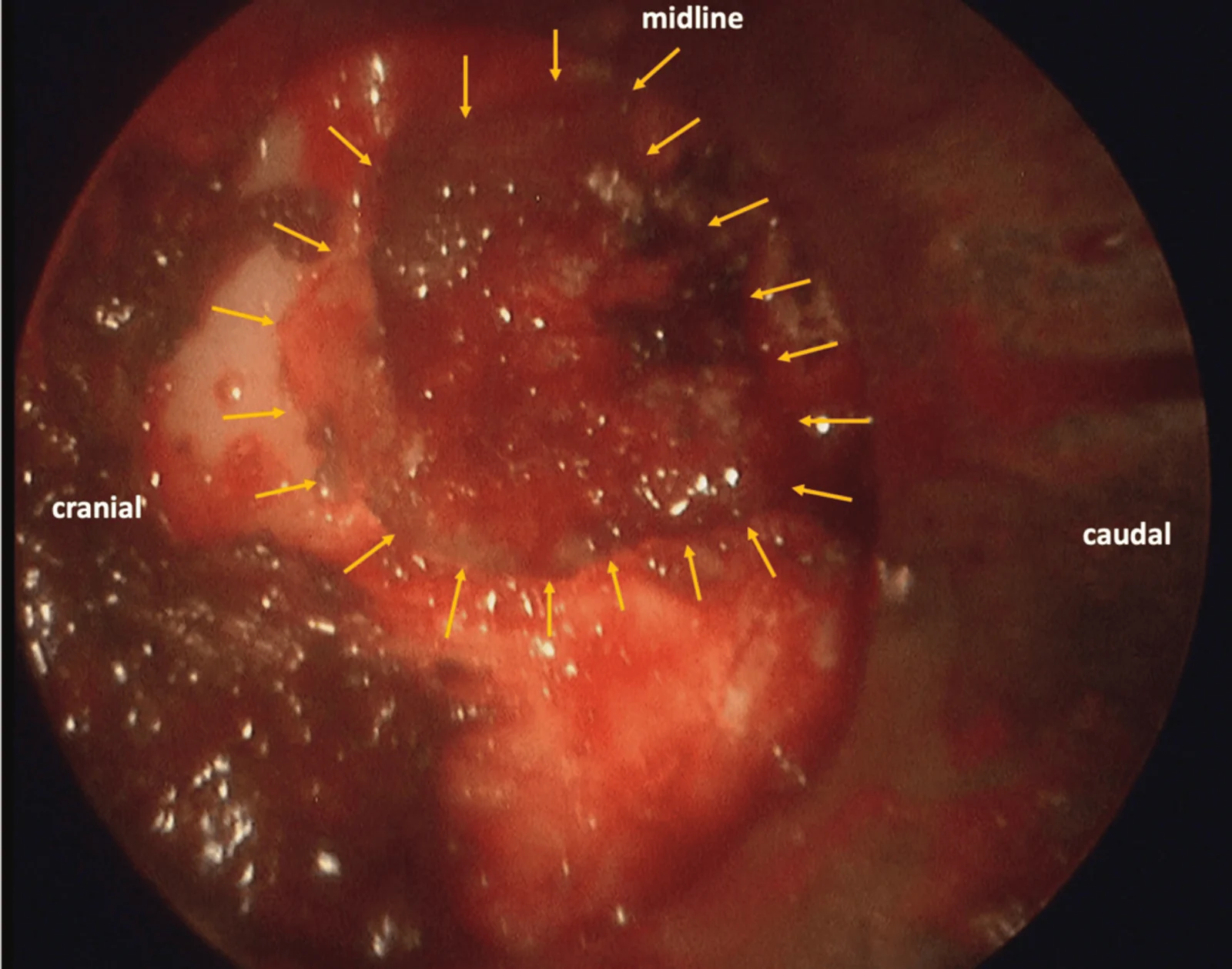
Intraoperative endoscopic findings Arrows indicate the location of the tumor

Intraoperative CT was then obtained to confirm the site for curettage, and the lesion in the lateral mass was curetted according to the preoperative plan. Access required resection of a limited portion of the facet joint, while part of the joint was preserved (Figure 3B). The excision did not extend toward the transverse foramen, and the vertebral artery was not exposed or manipulated. The spinal cord was protected by direct endoscopic visualization throughout curettage. A second intraoperative CT was obtained and confirmed that the extent of excision matched the preoperative surgical plan (Figures 3A-3D). Operative time was 99 minutes, with 60 mL of blood loss. CT was performed using a low-dose pediatric protocol; the radiation dose was not quantified.

**Figure 3:**
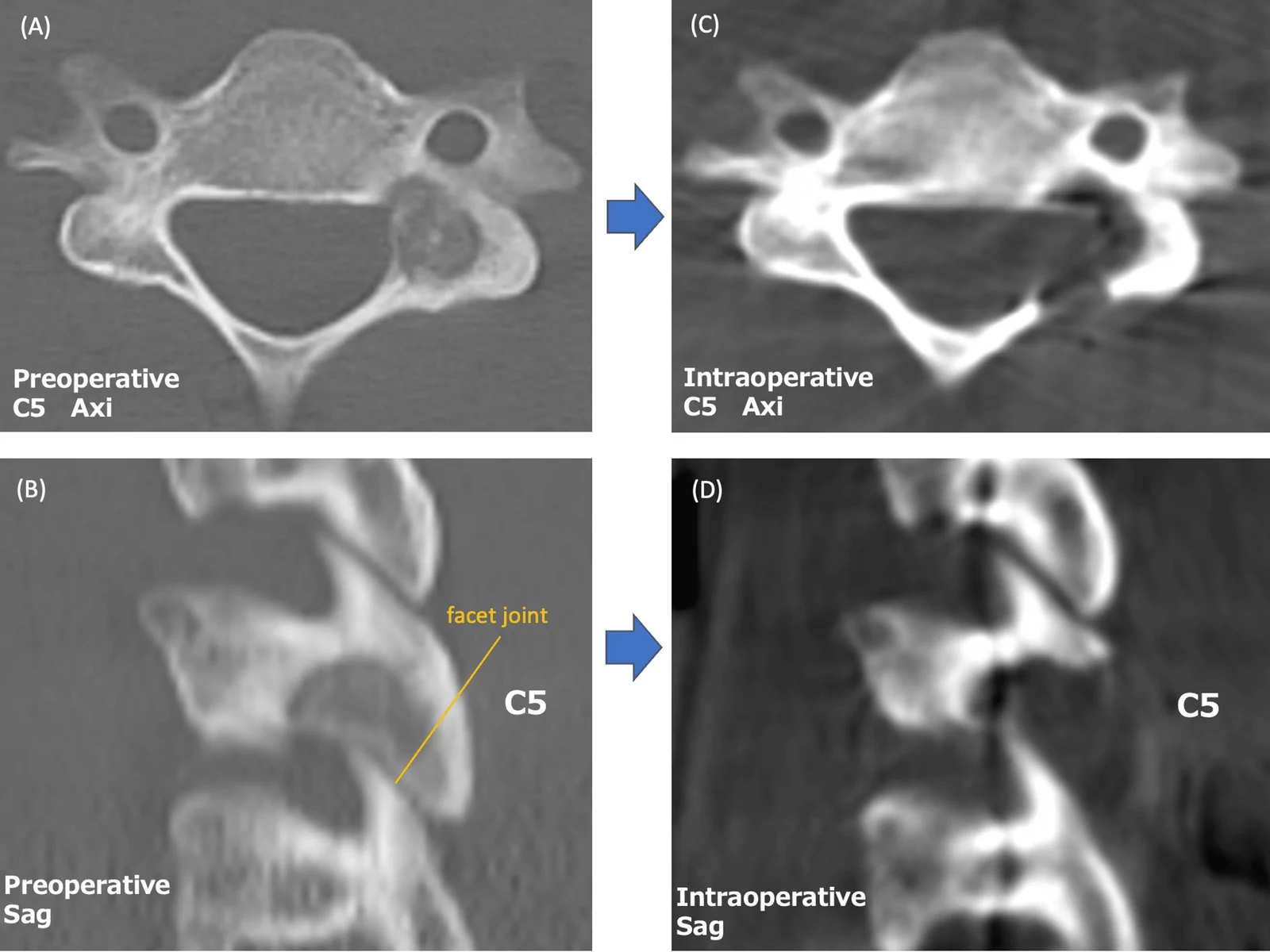
Comparison of preoperative and intraoperative CT scans Preoperative axial (A) and sagittal (B) CT of C5. Intraoperative axial (C) and sagittal (D) CT confirming that the extent of excision matched the preoperative surgical plan CT: computed tomography

Postoperative radiographs at one week showed no obvious abnormalities (C2-C7 Cobb angle, 16.2°) (Figure 4).

**Figure 4:**
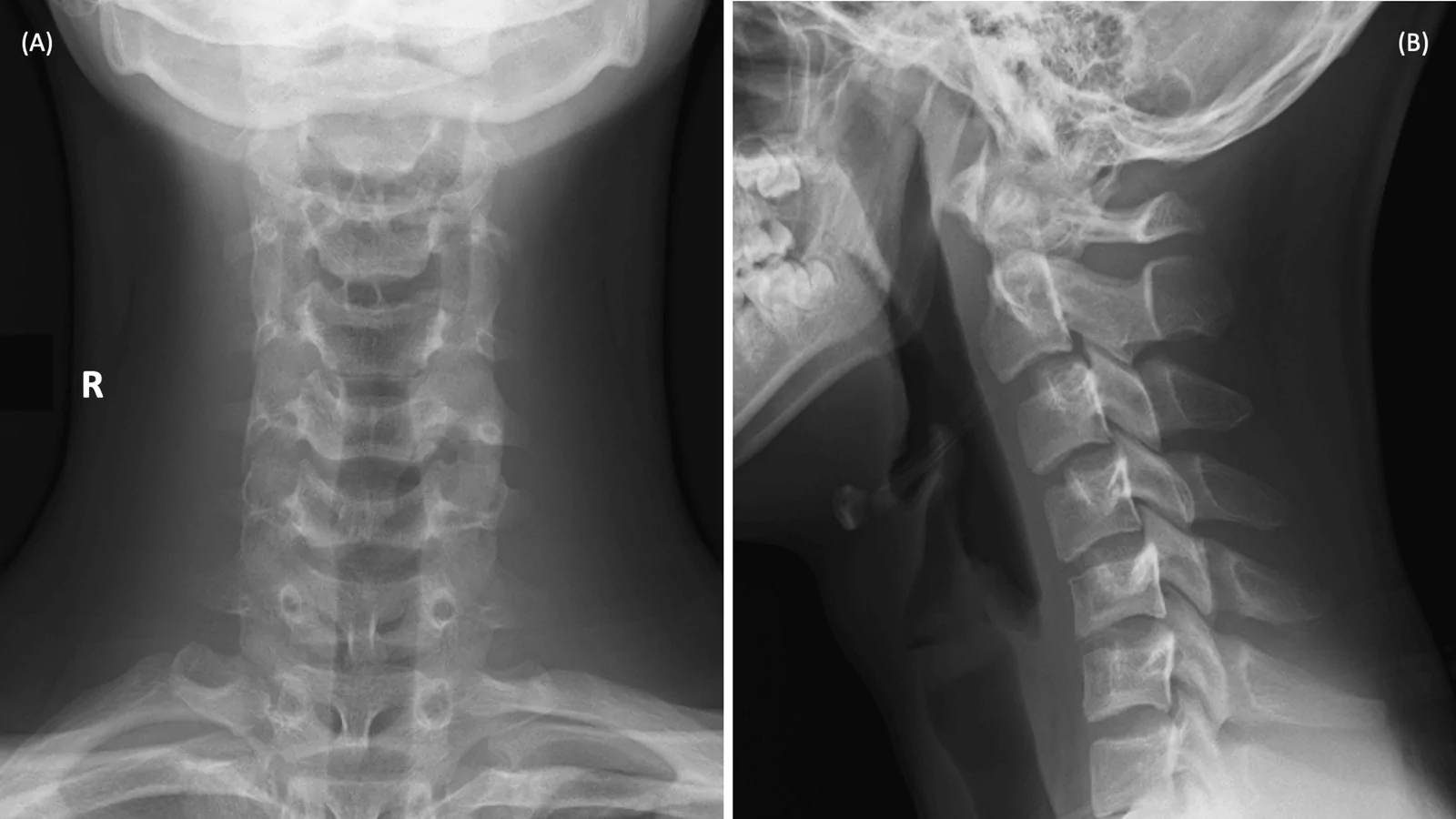
(A,B) Postoperative radiographs (one week), showing no obvious abnormalities (C2-C7 Cobb angle, 16.2°)

Histopathological examination revealed increased trabecular osteoid with calcification, with osteoblasts and spindle-shaped cells in the intertrabecular spaces; nuclear atypia was not conspicuous, and no fine lace-like bone was observed. These findings were not inconsistent with tissue from osteoid osteoma (Figure 5).

**Figure 5:**
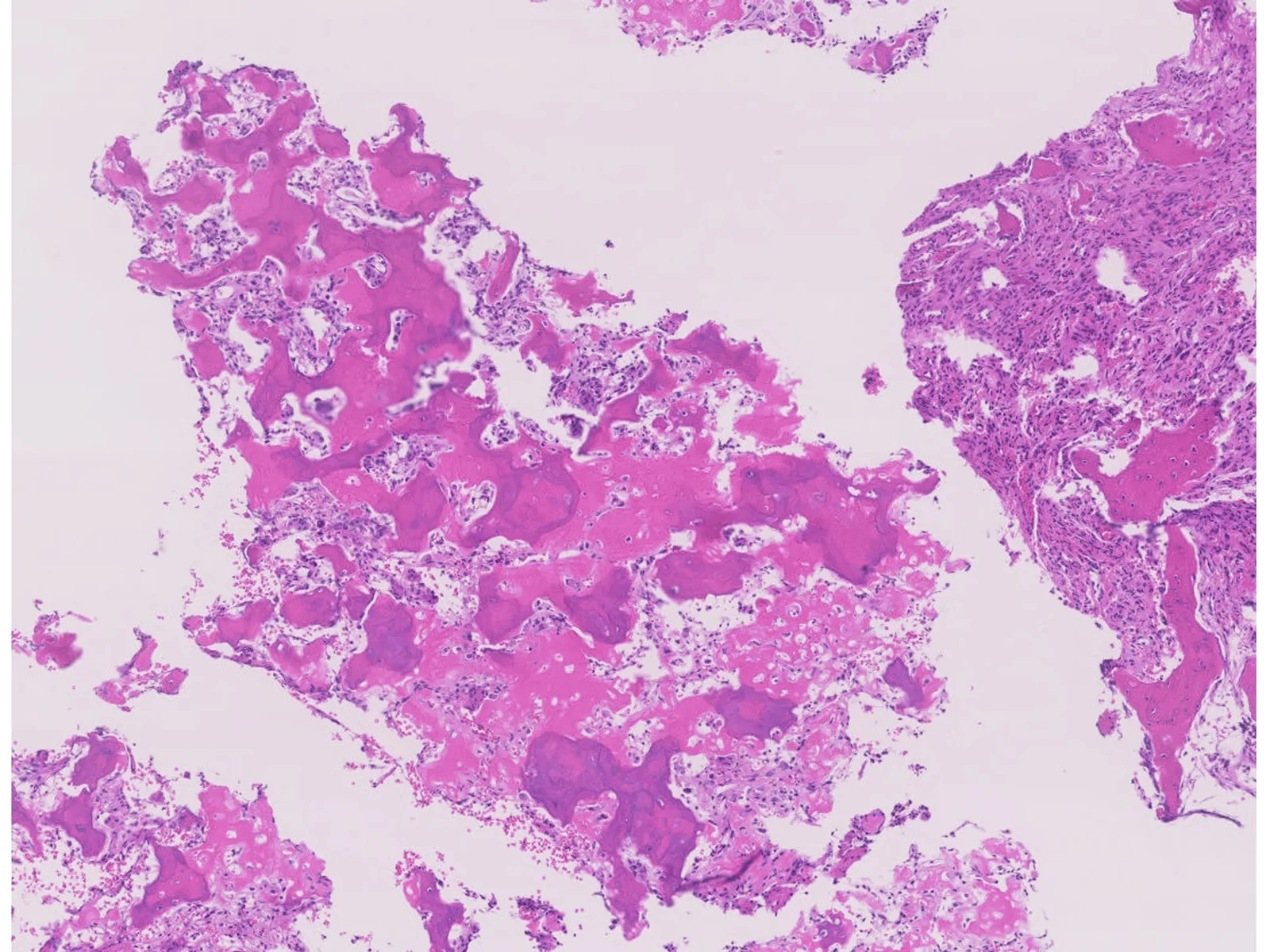
Pathological examination Histopathological examination (hematoxylin and eosin stain, ×10) showing increased trabecular osteoid with calcification, with osteoblasts and spindle-shaped cells in the intertrabecular spaces; nuclear atypia is not conspicuous, and no fine lace-like bone is seen

Left posterior neck pain resolved immediately after surgery. The patient was immobilized in a soft collar for one month. Range-of-motion stretching began at one week, jogging was permitted at one month, and contact sports, including basketball, were permitted at three months, coinciding with a tournament. No formal rehabilitation protocol was used; return to sports was allowed based on clinical findings, particularly resolution of pain. Follow-up radiographs and CT two years after surgery (Figure 6) showed no progressive spinal malalignment or spondylolisthesis (C2-C7 Cobb angle, 13.4°) and bone remodeling at the curettage site, with no evidence of recurrence.

**Figure 6:**
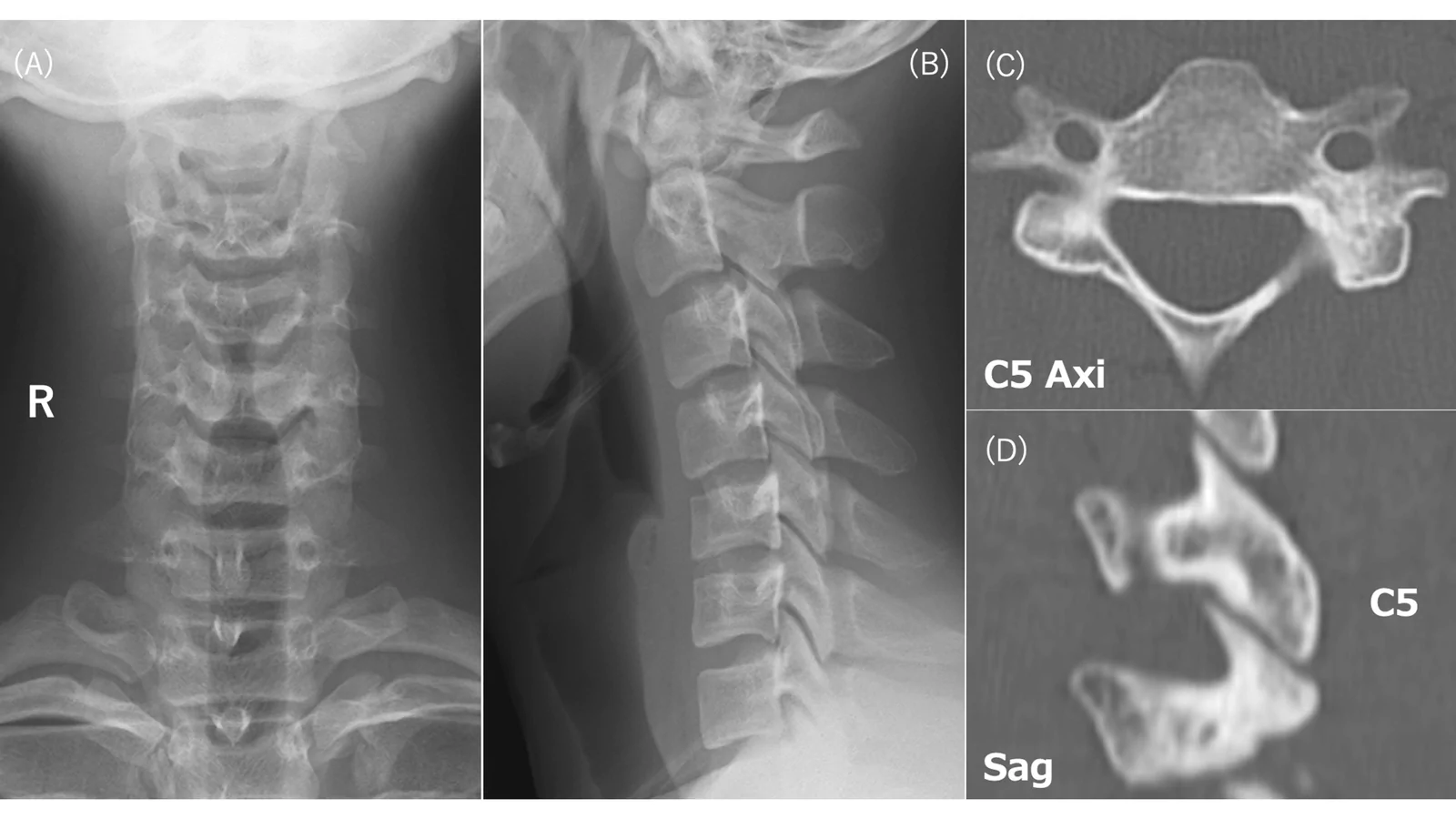
Postoperative imaging at two years (A,B) Radiographs showing no progressive spinal malalignment or spondylolisthesis (C2-C7 Cobb angle, 13.4°). (C,D) CT showing bone remodeling at the curettage site, with no evidence of recurrence

## Discussion

Osteoid osteoma is usually treated with NSAIDs, but surgical excision is chosen when NSAID treatment is ineffective. In recent years, CT-guided RFA has been reported to be effective in some cases [2]. However, percutaneous RF electrodes are generally recommended to remain at least 1 cm from major neural structures [7], and in the present case, the lesion was located only 3 mm from the spinal cord. Although intraoperative motor-threshold monitoring has been reported to allow safe RFA closer to neural structures [8], this technique was not used in our case, and RFA was therefore not considered.

Complete excision of the nidus is essential to prevent recurrence, and incomplete excision has been reported to be a major cause of recurrence, with recurrence rates of approximately 7% in spinal lesions [9]. However, excessive bone excision in the spine may lead to instability, and therefore, achieving a balance between adequate tumor excision and minimal invasiveness remains a challenge.

Although there have been few reports of endoscopic excision of osteoid osteoma in the cervical spine, it has been suggested to minimize tissue damage and reduce postoperative pain and deformity [4]. Furthermore, remodeling of the curettage site is expected in sufficiently young patients [10]. In the present case, the endoscopic approach allowed targeted exposure, and bone removal was limited to the planned area. Bone remodeling was observed at the curettage site during follow-up (Figure 6).

Because the excision in this case was limited to the lamina and a portion of the facet joint, with the facet joint largely preserved, fusion was not required. Facetectomy exceeding 50% has been reported to cause pronounced increases in angular motion and disc stress in biomechanical studies [3]; although the extent of facet resection in this case was not quantified, the C2-C7 Cobb angle changed only modestly across the perioperative period (12.5° preoperatively, 16.2° at one week, and 13.4° at two years postoperatively), without progressive kyphotic deformity, suggesting that segmental stability was preserved. Avoiding unnecessary fusion is desirable in young patients because it preserves segmental motion; however, this single case does not establish an advantage of endoscopic excision over other treatment approaches.

On the other hand, endoscopic excision may cause incomplete excision of the tumor or excessive excision of the articular process because of insufficient surgical field of view [5,6]. By combining intraoperative CT with endoscopic excision, we were able to confirm that the extent of excision matched the preoperative surgical plan, helping overcome this limitation of endoscopic surgery. There has been a report of percutaneous excision of osteoid osteoma using intraoperative CT [11]. Although intraoperative CT-guided excision of osteoid osteoma has been reported previously, to our knowledge, this is the first report describing its combination with endoscopic excision. Because the CT acquisitions in this case used a low-dose pediatric protocol without formal dose quantification, the specific radiation exposure could not be reported; quantifying and minimizing cumulative radiation dose remains an important consideration when using intraoperative CT in pediatric patients, particularly given the additional intraoperative acquisitions required in this case.

In the present case, MRI showed bone marrow edema and soft tissue signal change around the lesion; such findings, well recognized in osteoid osteoma, can mimic more aggressive-appearing lesions such as osteoblastoma or osteomyelitis, both of which were part of the differential diagnosis in this case. Reliance on MRI alone could therefore bias the surgeon toward unnecessarily extensive surgery. CT, in contrast, delineates the nidus more precisely and was central to our diagnostic and surgical planning, helping to avoid this bias.

## Conclusions

This case suggests that endoscopic excision combined with intraoperative CT may be a feasible treatment option for cervical osteoid osteoma located adjacent to the spinal cord. Intraoperative CT enabled precise localization of the nidus and confirmation that the extent of excision matched the preoperative surgical plan, thereby helping overcome the limited visualization inherent to endoscopic surgery. The patient experienced immediate pain relief, returned to sports within three months, and showed favorable bone remodeling with no recurrence, progressive spinal malalignment, or spondylolisthesis during two years of follow-up. To our knowledge, this is the first report describing the combination of endoscopic excision and intraoperative CT for osteoid osteoma of the cervical spine. Although this single-case experience does not establish the general safety, superiority, or reproducibility of this technique, it demonstrates the feasibility of the approach in this patient. Further accumulation of cases is warranted to confirm its clinical utility and safety.
